# Molecular Imaging: a Novel Tool To Visualize Pathogenesis of Infections *In Situ*

**DOI:** 10.1128/mBio.00317-19

**Published:** 2019-10-29

**Authors:** Oren Gordon, Camilo A. Ruiz-Bedoya, Alvaro A. Ordonez, Elizabeth W. Tucker, Sanjay K. Jain

**Affiliations:** a Division of Infectious Diseases, Department of Pediatrics, Johns Hopkins University School of Medicine, Baltimore, Maryland, USA; b Center for Infection and Inflammation Imaging Research, Johns Hopkins University School of Medicine, Baltimore, Maryland, USA; c Department of Anesthesiology and Critical Care Medicine, Johns Hopkins University School of Medicine, Baltimore, Maryland, USA; University of Texas Health Science Center at Houston

**Keywords:** heterogeneity, infectious disease, molecular imaging, pathogenesis, positron emission tomography

## Abstract

Molecular imaging is an emerging technology that enables the noninvasive visualization, characterization, and quantification of molecular events within living subjects. Positron emission tomography (PET) is a clinically available molecular imaging tool with significant potential to study pathogenesis of infections in humans. PET enables dynamic assessment of infectious processes within the same subject with high temporal and spatial resolution and obviates the need for invasive tissue sampling, which is difficult in patients and generally limited to a single time point, even in animal models. This review presents current state-of-the-art concepts on the application of molecular imaging for infectious diseases and details how PET imaging can facilitate novel insights into infectious processes, ongoing development of pathogen-specific imaging, and simultaneous *in situ* measurements of intralesional antimicrobial pharmacokinetics in multiple compartments, including privileged sites. Finally, the potential clinical applications of this promising technology are also discussed.

## Abstract (precis)

Molecular imaging is an emerging technology that enables the noninvasive visualization, characterization, and quantification of molecular events within living subjects. Positron emission tomography (PET) is a clinically available molecular imaging tool with significant potential to study pathogenesis of infections in humans.

## INTRODUCTION

Noninvasive molecular imaging is a powerful clinical tool for the early diagnosis and monitoring of various disease processes. Next-generation molecular imaging promises unparalleled opportunities for visualizing infections, since molecular and cellular alterations occur earlier than structural changes in a pathological process. This rapidly developing technology has already become an essential tool in the field of oncology, with similar potential for infectious diseases (1). Currently there are several available molecular imaging techniques. Optical imaging with bioluminescent or fluorescent biomarkers is widely used and has excellent sensitivity. However, it is generally two-dimensional and importantly has limited depth penetration (<1 cm), restricting its use to small animal models and surgical or endoscopic procedures in patients (2). Novel methodologies recently developed, significantly enhance light microscopy capabilities. The CLARITY technique replaces target tissue lipids with a water-based gel that renders the tissue optically transparent enabling intact-tissue staining in nonsectioned tissue (3) and enabled high-resolution and detailed three-dimensional *in situ* imaging, however, as more traditional methods, these methods relay on invasive tissue acquisition. Conversely, nuclear-medicine-based molecular imaging utilizes “tracers” or “probes” labeled with high-energy emission radionuclides, which can be used to target specific molecular pathways deep inside the body (4, 5). Three-dimensional spatial localization of biomarkers in nuclear medicine techniques is determined by measuring the source of the radionuclide attached to the biomarker. Among the available molecular imaging techniques, positron emission tomography (PET) is highly sensitive (pmol/liter) and can be used to visualize a variety of *in vivo* biological processes (6). PET is often coregistered with conventional imaging such as computed-tomography (CT) or magnetic resonance imaging (MRI) for anatomic reference (7). Advancements in technology, such as whole-body PET (8), enable exquisite sensitivity (40×), increasing the clinical utility of PET. Nonnuclear and clinically available MRI-based molecular imaging approaches such as magnetic resonance spectroscopy (MRS) are also able to provide detailed structural, functional, and metabolic information utilizing endogenous or exogenous contrast agents, although with a lower sensitivity than PET. Finally, ultrasound and photoacoustic imaging are also being developed for molecular imaging applications with promise for future applications to infections.

Molecular PET imaging allows the integration of molecular and physiological data with anatomical information in individual patients. In oncology, clinical molecular PET imaging enables early detection, real-time therapeutic monitoring, and the ability to streamline drug development (9). PET utilizing ^18^F-labeled fluorodeoxyglucose (^18^F-FDG), a glucose analog that is selectively taken up by cells with a high rate of glucose metabolism, is a valuable clinical tool for predicting tumor response to treatment and patient survival (10). However, ^18^F-FDG is nonspecific and accumulates in tissues with increased metabolic activity regardless of the underlying pathology (i.e., cancer, inflammation, infection). Therefore, target-specific PET probes for cancer are being developed to allow for a more specific diagnosis (11). In drug development, molecular PET imaging is particularly useful in target validation, whole-body target expression and heterogeneity, whole-body drug distribution, pharmacokinetics (PK) (e.g., drug penetration into privileged sites such as the central nervous system [CNS] penetration), and pharmacodynamic (PD) effects (12). Other areas in medicine also use molecular PET imaging. For instance, PET is used for monitoring autoimmune and inflammatory diseases and vasculitis (13). In cardiology, PET can evaluate cardiac metabolism (i.e., myocardial viability, perfusion, inflammation) in heart failure (14). Treatment of patients with cardiovascular disease increasingly incorporates PET into management algorithms due to its use in detecting atherosclerosis, thrombosis, and myocardial infarction (15). Finally, molecular imaging for the diagnosis and management of infectious diseases is gaining momentum with technological advancements and a growing clinical need for holistic and individualized information for patient care, not feasible with other current technologies.

## UNDERSTANDING DISEASE PATHOGENESIS *IN SITU*

**FIG 1:**
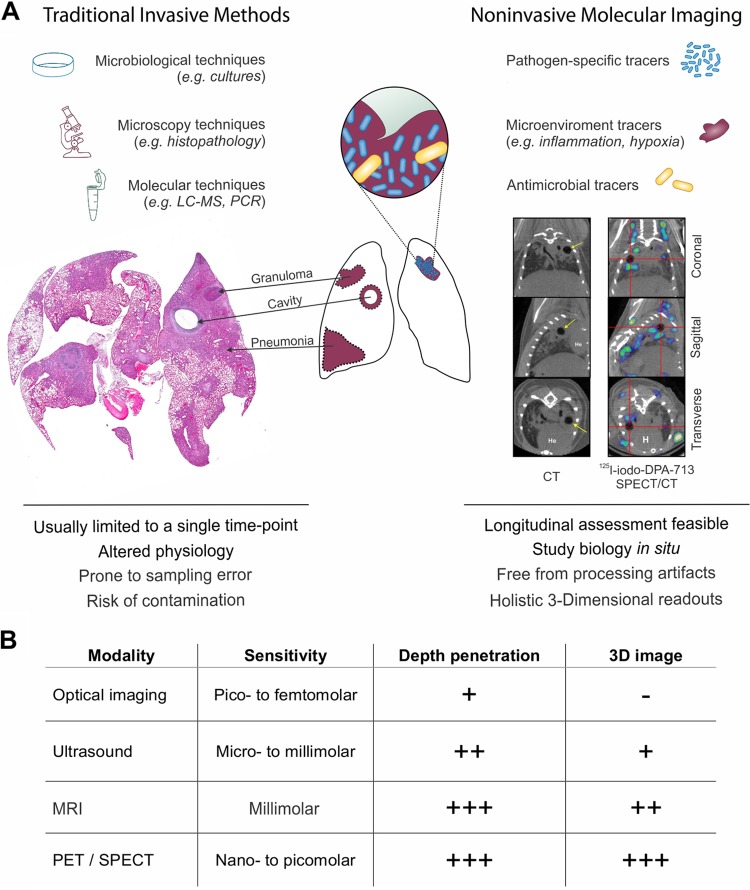
Molecular imaging tools. (A) Traditional tools used to study infections such as microbiology, microscopy, and molecular techniques (e.g., PCR and mass spectrometry), require tissue excision, which is prone to contamination and sampling bias, and the tools are also generally limited to a single time point. Molecular imaging can address some of these limitations and complement traditional tools. Histology and imaging were adapted from Ordonez et al. (34, 86). (B) Comparison of the commonly available molecular imaging techniques. PET, positron emission tomography; SPECT, single photon emission computed tomography; CT, computed tomography.

Understanding the pathogenesis of infectious diseases is essential in the development and assessment of novel therapeutics. It would be particularly useful if the same tools utilized in preclinical models could be also translated into the clinic and thus help in the standardization of the readouts. PET imaging of live animals or patients to study infections overcomes several fundamental limitations of current laboratory techniques which are generally invasive and rely on tissue resection. Molecular imaging provides holistic three-dimensional readouts and allows the study of biology *in situ*, with relatively unaltered physiology and lack of processing or other artifacts that can occur during resection and analysis of tissues (Fig. 1). It also allows longitudinal profiling in the same subject at several time points to understand the changes in lesion pathology over time, while reducing subject-to-subject variability.

### Spatial heterogeneity.

It is increasingly being recognized that many different infectious lesions with distinct bacterial burdens, antimicrobial exposures, and local biology can coexist in the same host (16–19). Molecular imaging can measure inter- and intrasubject heterogeneity at a lesion, organ, or whole-body level. Molecular PET imaging can also characterize these local heterogeneous microenvironments for pathogen dynamics, immune response, and environmental cues. For example, hypoxia is considered to be a major determinant of bacterial persistence in human tuberculosis (TB) (20). Therefore, Harper et al. (21) used copper-64(II)-diacetyl-bis(N4-methyl-thiosemicarbazone) (^64^Cu-ATSM), a PET tracer used to detect hypoxic lung lesions in a mouse model of TB and confirmed using postmortem analyses. While no tracer accumulation was noted in nonhypoxic TB lesions in acutely infected or in control mice without lesions, a progressive, time-dependent tracer accumulation was noted in chronically infected mice, which are considered hypoxic. These findings were subsequently confirmed in TB patients using ^18^F-fluoromisonidazole (^18^F-FMISO) PET imaging (22). In another study, Davis et al. (23) used single-photon emission computed tomography (SPECT) to detect and localize an engineered Mycobacterium tuberculosis strain where a bacterial thymidine kinase (TK) was introduced under the control of a strong mycobacterial promoter. TK phosphorylates 1-(2-deoxy-2-fluoro-β-d-arabinofuranosyl)-5-^125^I-iodouracil (^125^I-FIAU), a nucleoside analog, leading to trapping and accumulation of ^125^I-FIAU in the M. tuberculosis Phsp60 TK strain. Thus, bacteria were specifically and noninvasively detected in experimentally infected animals demonstrating heterogeneous bacterial burdens in visible TB lesions (23). Infection dynamics are closely related to the host immune response. In a well-established nonhuman primate model of tuberculosis (24), Martin et al. used genome-encoded barcodes to uniquely tag individual M. tuberculosis bacilli and quantitatively track the trajectory of the infecting bacterium (25). By coupling this tagging strategy with ^18^F-FDG PET/CT of lung pathology in macaques, they demonstrated that a subset of TB lesions, distinguishable by imaging features, were responsible for the majority of bacterial dissemination (25). ^18^F-FDG PET has also been employed to monitor the heterogeneity of the host metabolic responses. In a nonhuman primate model of cerebral malaria, ^18^F-FDG PET demonstrated decreased cerebral metabolic activity. A diffuse and heterogeneous reduction of metabolic activity in the frontal and temporal lobes was noted prior to evidence of neuropathological findings (26).

### Temporal monitoring.

**FIG 2:**
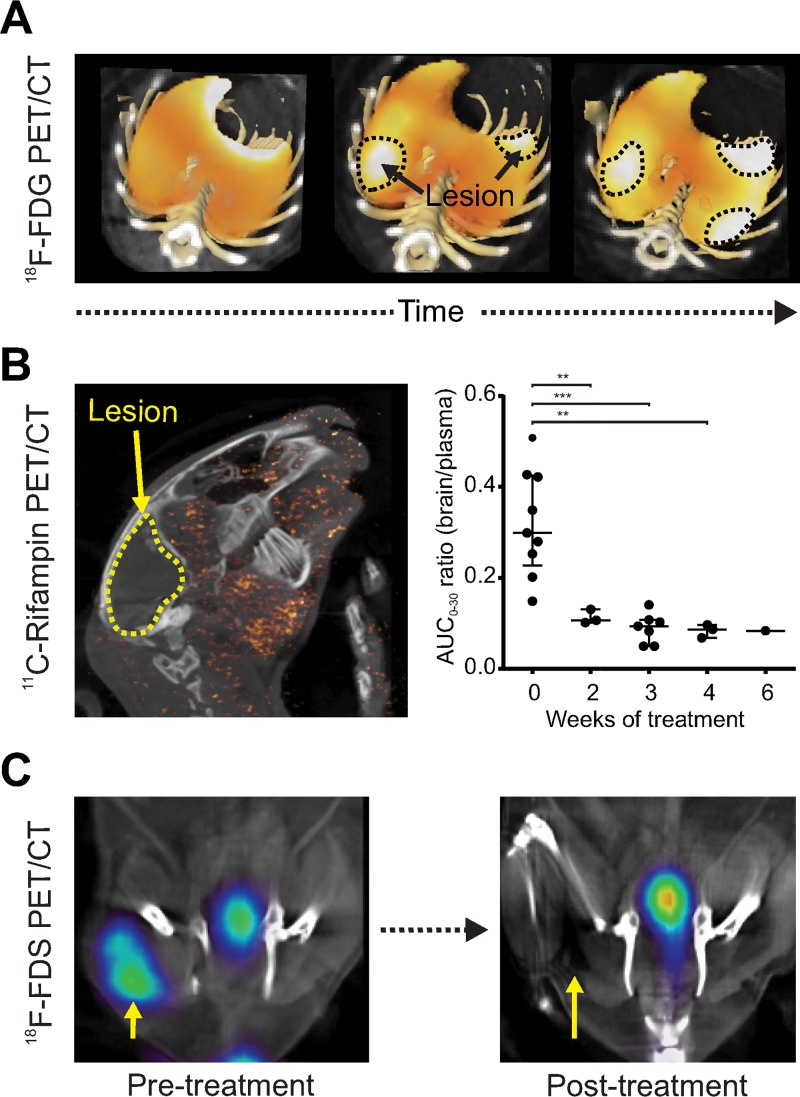
Temporal monitoring. Imaging allows for repeat measurements to quantify temporal changes in the same subject. (A) Serial monitoring of individual TB pulmonary lesions in the same mouse demonstrates dynamic and independent evolution. (B) Serial ^11^C-rifampin PET in a rabbit model of TB meningitis demonstrates spatially heterogeneous brain penetration that rapidly decreased as early as 2 weeks into treatment (adapted from Tucker et al. [29]). (C) ^18^F-FDS PET performed before and after initiation of antimicrobial treatment in a murine model of E. coli myositis can rapidly monitor treatment efficacy, demonstrating a PET signal proportionate to the bacterial burden. This method can also be used to detect therapeutic failures due to infections with multidrug-resistant, extended-spectrum β-lactamase (ESBL)-producing E. coli (adapted from Weinstein et al. [36]).

PET imaging allows for repeated measurements to quantify temporal changes in the same subject. Dormant bacteria are commonly believed to inhabit established TB lesions, although this is controversial (27). Nonetheless, the spatial location of dormant bacteria has never been demonstrated experimentally in live hosts, and their precise location still remains elusive. Therefore, Murawski et al. utilized sequential ^18^F-FDG PET/CT to monitor the spatial and temporal evolution of individual pulmonary TB lesions in experimentally infected mice over the course of TB treatment and subsequent development of relapse (28). They discovered that although several new lesions arose *de novo* during reactivation TB within lungs regions with no lesions prior to TB treatment, suggesting that dormant bacteria may also reside outside established lesions. Similarly, serial PET/CT in M. tuberculosis-infected macaque lungs and a rabbit model of TB meningitis also demonstrated that individual TB lesions are dynamic and change independently during infection with different drug penetration and with lesions regressing and egressing in the same host (29, 30). Figure 2A demonstrates the utility of ^18^F-FDG PET/CT to follow M. tuberculosis pulmonary lesions in a murine model of pulmonary TB.

Repeat measurements can also be used to monitor treatment and provide prognostic information. For example, ^18^F-FDG PET was successfully used to evaluate the bactericidal activity of multidrug treatments in mice (31). In cynomolgus macaques with latent TB, increased ^18^F-FDG PET pulmonary activity or extrapulmonary involvement prior to measures that induce reactivation TB ([tumor necrosis factor alpha TNF-α] neutralizing antibody) predicted reactivation TB with high accuracy (32). Activated macrophages are the key components of TB-associated inflammation. Foss et al. demonstrated that radioiodinated DPA-713, a synthetic ligand of the translocator protein (TSPO) which is highly upregulated in activated microglia and macrophages, is selectively retained within macrophages and phagocytic cells in pulmonary TB lesions (33). In a subsequent study comparing ^125^I-DPA-713 SPECT with ^18^F-FDG PET in a mouse model of pulmonary TB, ^125^I-DPA-713 SPECT was found to be a better predictor than ^18^F-FDG PET of early bactericidal activities of TB treatments (34). ^124^I-DPA-713 PET has also been utilized successfully to image neuro-inflammation in a rabbit model of TB meningitis (35). Pathogen-specific PET tracers have also been utilized to rapidly monitor response to antimicrobial treatments and detect therapeutic failures associated with drug-resistant organisms (36, 37). In simian immunodeficiency virus (SIV)-infected rhesus macaques ^64^Cu-labeled SIV Gp120-specific antibody was used for PET imaging and was able to trace viral dynamics following antiretroviral treatment (ART) identifying reservoirs of the virus even in monkeys controlling the infection (38). Complementing this with *in vivo* tracing of CD4 T lymphocytes, could provide a comprehensive evaluation of the response to treatment (39).

### Molecular imaging to study antimicrobial PK/PD *in vivo*.

While antimicrobials are among the most commonly prescribed drugs, up to 50% are inappropriately utilized or not optimized for efficacy (40). Antibacterial efficacy is determined by the susceptibility of the microorganism to the antimicrobial, which is a feature of the organism and commonly assayed using the MIC (41). However, the MIC does not account for the heterogeneous *in vivo* microenvironments that may change the bacterial susceptibility to the antibiotic as well as antimicrobial exposures achieved at infection sites *in vivo*. While MIC data in combination with clinical studies have been used to determine antimicrobial breakpoints and antimicrobial dosing (42), they are based on measurements of antimicrobial concentrations in plasma, which do not always accurately correlate with intralesional or target tissue concentrations, nor take into account the heterogeneity of different infected tissues in the same host (43). A low concentration of the antimicrobial in the infected tissue may lead to treatment failure, require the need for prolonged treatment durations, and promote the emergence of drug-resistant organisms. Conversely, unnecessarily high antimicrobial concentrations can lead to toxicities, organ injury, drug intolerance, and noncompliance (44). There is an urgent unmet need to improve the current knowledge of antibiotic intralesional PK/PD properties to optimize antimicrobial use and facilitate new drug development (4). Liquid chromatography-tandem mass spectrometry (LC-MS/MS) and matrix-assisted laser desorption ionization (MALDI) are promising techniques that can provide detailed intralesional biodistribution but require invasive tissue sampling (45). However, these techniques are prone to sampling errors and generally limited to single time points, precluding the ability to measure PK parameters such as area under the concentration-time curve (AUC) or changes in drug concentrations with treatment or progression of disease (46).

Whereas the use of PET for PK/PD assessments in oncology has facilitated efforts for drug discovery and clinical translation (47), its application for infectious diseases is still an emerging field (48). PET utilizing radiolabeled antimicrobials provides a noninvasive solution to these challenges. For example, rifampin is a key first-line TB antibiotic required for sterilization and also used to treat infections due to other bacteria such as Staphylococcus aureus. However, even after 50 years of clinical use, we still do not know how to optimally dose rifampin. Recently, Tucker et al. utilized ^11^C-rifampin, a radiolabeled analog of rifampin to optimize treatments for TB meningitis (29). Sequential ^11^C-rifampin PET in a rabbit model of TB meningitis demonstrated limited and spatially heterogeneous brain penetration that rapidly decreased as early as 2 weeks into treatment (Fig. 2B). Similarly, in pulmonary TB, rifampin exposure was lower in infected lesions and paradoxically lowest in cavitary walls, even though cavities have an extremely high bacterial burden (10^7^ to 10^9^) and thus are a risk factor for transmission (49, 50). These PET data support the ongoing efforts to develop high-dose rifampin-based regimens to treat TB—especially those in privileged compartments (e.g., TB meningitis or pulmonary cavitary TB) with limited and variable antimicrobial penetration (51, 52). Similar studies have been performed to evaluate intralesional concentration of existing or new antimicrobial (e.g., bedaquiline) to optimize antimicrobial treatments (53, 54).

## DEVELOPMENT OF PATHOGEN-SPECIFIC IMAGING TOOLS

Current radiopharmaceuticals to image infections rely on nonspecific pathophysiological consequences of infection, such as increased capillary permeability, vasodilation, and hyperemia, as well as adaptation of local metabolism (55). Similarly, while ^18^F-FDG PET is a valuable tool for the diagnosis and management of infectious diseases (56), it is not specific for infection and cannot differentiate infection from other disease processes, such as inflammation or cancer. Development of pathogen-specific tracers has been challenging and, in the past, demonstrated variable results. Many pathogen-specific tracers have relied on antimicrobial-derived radiopharmaceuticals (e.g., ^99m^Tc-ciprofloxacin [57]), or antimicrobial peptides with variable results (58), likely due to lack of bacterial accumulation that is needed to reliably detect infections from the background tissues (59).

### Bacterium-specific PET imaging.

Mainstay clinical microbiology uses differential bacterial metabolism, such as selective growth media, to differentiate bacteria in the microbiology laboratory (60). Therefore, recent attempts utilizing small molecules selectively metabolized by bacteria but not by mammalian cells hold promise. Ordonez et al. presented a systematic discovery approach to identify and develop novel bacterium-specific PET tracers based on selective metabolism (61). They performed an *in silico* screen of 961 radiolabeled small molecules followed by bacterial uptake assays of promising candidates and identified several potential bacterium-specific imaging tracers such as *para*-aminobenzoic acid (PABA), which accumulated in all bacterial species tested, d-mannitol, which accumulated selectively in Gram-negative and Gram-positive bacteria but not mycobacteria, and d-sorbitol, which accumulated selectively in Gram-negative *Enterobacteriaceae* (Escherichia coli, Klebsiella pneumoniae, *Yersinia* spp., *Enterobacter* spp., etc.) which is the largest family of bacterial pathogens in humans. Together, these afford a set of tools for the differential imaging of bacteria *in vivo*.

Several investigators are utilizing this approach to develop bacterium-specific tracers. For instance, maltose and maltodextrin are polysaccharides that are incorporated with high specificity using the maltose-maltodextrin transport system present in multiple Gram-negative as well as Gram-positive bacteria but not in mammalian cells (62). Several generations of tracers have been developed to target the maltodextrin transporter system (63, 64). Most recently, 6″-^18^F-fluoromaltotriose (65) was shown to selectively accumulate in an E. coli myositis and Pseudomonas aeruginosa wound infection mouse models. Similarly, sorbitol, a sugar alcohol, is selectively taken up via surface transporters, phosphorylated and further metabolized by *Enterobacteriaceae* (61). *In vitro* uptake of ^18^F-fluorodeoxysorbitol (^18^F-FDS), which can be easily synthesized from ^18^F-FDG, demonstrated that the tracer accumulated ∼1,000-fold more in *Enterobacteriaceae* than in mammalian cells. Moreover, ^18^F-FDS PET was able to specifically detect and differentiate live *Enterobacteriaceae* (infected lesion) from sterile inflammation (heat-killed bacteria) in a murine thigh myositis model as well as other animal models of infection (36, 66). Other recently developed bacterium-specific imaging agents include ^11^C-PABA, 2-^18^F-PABA, and ^18^F-fluoropropyl-trimethoprim (^18^F-FPTMP), which target the bacterial folate pathway (37, 67, 68), radio-analogs of d-amino acids that are incorporated into the bacterial cell wall (69), and siderophore-derived agents (70). ^18^F-FDS has been administered to humans and found to be safe and well-tolerated (71).

Since successful treatment often leads to rapid killing or inactivation of the pathogen much earlier than the resolution of inflammation or tissue destruction, pathogen-specific imaging also holds promise for the rapid detection of therapeutic responses. Antimicrobial resistance can be identified because the metabolic pathways utilized by many bacterium-specific tracers are highly conserved in susceptible and multidrug-resistant organisms (MDROs) (61). Therefore, based on the differential response to antibiotics when treating susceptible versus resistant bacteria, many bacterium-specific PET imaging tracers were able to rapidly identify infections due to MDROs without any invasive procedures (Fig. 2C) (36, 37), with potential for clinical translation.

### Virus-specific PET imaging.

Attempts have also been made to image viral infections by utilizing antiviral molecules such as acyclovir and its derivatives (72). For example, Buursma et al. used 9-[(1-^18^F-fluoro-3-hydroxy-2-propoxy)methyl]guanine (^18^F-FHPG), a derivative of ganciclovir, in which one hydroxyl group is replaced with a radioactive fluorine atom (73). ^18^F-FHPG is selectively phosphorylated by the herpes simplex virus (HSV) TK and becomes trapped within infected cells. This method was utilized to visualize HSV-affected brain regions in a rat model of encephalitis (73). ^64^Cu-labeled simian immunodeficiency virus (SIV) Gp120-specific antibody has also been utilized to study the viral dynamics and localization of SIV in viremic and antiretroviral therapy-treated macaques using PET (38). In viremic macaques, PET signal was detectable in the gastrointestinal and respiratory tract, lymphoid tissues, and reproductive organs. In contrast, antiretroviral-treated (aviremic) macaques had much lower signals but which were still detectable in colon, select lymph nodes, small bowel, nasal turbinates, the genital tract, and lung. In elite controllers, the PET signal was localized to the small bowel, select lymphoid areas, and the male reproductive tract. Novel, virus-specific PET tracers could also be developed by targeting specific metabolic process required for the viral replication cycle (72).

### Fungus-specific PET imaging.

Fungal pathogens, including yeast such as *Candida* sp. and molds such as aspergillosis, cause significant morbidity and mortality, especially in vulnerable hosts that are immunosuppressed. However, diagnosing these infections may be challenging because they are particularly difficult to isolate, grow, and manipulate in the laboratory. ^99m^Tc-fluconazole as well as ^99m^Tc-labeled peptides have been utilized to detect Candida albicans infections in mice but not found to be optimal tracers (74). Radiolabeled *Aspergillus*-specific monoclonal antibodies (MAbs) also hold potential to provide a more specific tool for diagnosis (75). The *Aspergillus*-specific mouse MAb mJF5 and its humanized derivative hJF5 are directed at the extracellular galacto-mannoprotein antigens produced by all clinically relevant *Aspergillus* species (76). Administration of ^64^Cu-DOTA-labeled MAb mJF5 to neutrophil-depleted A. fumigatus-infected mice allowed specific localization of lung infections when imaged with PET (77). Siderophores produced by fungi, such as triacetylfusarinine C (TAFC), which are selective for mold, have also been used as a pathogen-specific imaging strategy for aspergillosis. Sequential PET/CT with ^68^Ga-TAFC in a rat model of invasive pulmonary aspergillosis could detect infection much earlier than conventional technique (78), although no subsequent studies have been reported using this tracer. Recently, Lindeman et al. also reported the use of an advanced MRI technique, chemical exchange saturation transfer (CEST) to measure the extracellular pH of tissues *in vivo* in murine models (79). While not specific for fungi, they demonstrated that the pH of lung adenocarcinoma lesions were consistently lower than those in the granulomas (lesions) developed due to coccidioidomycosis, suggesting an interesting methodology to differentiate these distinct pathologies that otherwise can present with similar clinical and conventional imaging findings.

## CLINICAL TRANSLATION

PET is becoming a routine clinical tool and is proliferating in the United States and abroad, although its application to infectious disease is mostly limited to ^18^F-FDG PET. For example, ^18^F-FDG PET in patients with pulmonary TB demonstrated nonresolving and intensifying lesions that correlated with the presence of M. tuberculosis mRNA in patients’ sputa, even following treatment and cure (80). This indicates that even apparently curative pulmonary TB treatment may not eradicate all organisms in many patients, which was a novel finding. In a cohort of patients with multidrug-resistant TB treated with second-line TB therapy for 2 years, quantitative changes in ^18^F-FDG uptake 2 months after starting treatment were associated with long-term outcomes, offering valuable early prognostic information (81). Similarly, early pathological metabolic activity on ^18^F-FDG PET, before initiation of antiretroviral therapy (ART), was associated with subsequent development of HIV-related immune reconstitution inflammatory syndrome (IRIS) after initiation of ART (82).

However, there is need for the development and clinical translation of more specific imaging tracers for infections. For example, ^124^I-DPA-713, a novel tracer for macrophage-associated inflammation, has recently been translated to the clinic. ^124^I-DPA-713 PET studies in healthy volunteers demonstrate that ^124^I-DPA-713 clears rapidly from the lungs, with predominantly hepatic elimination, and is safe and well tolerated in healthy adults (83). Human studies utilizing ^11^C-rifampin PET to study antimicrobial distribution and optimize treatments for TB meningitis (29) and pulmonary TB (49) have also been performed recently. Bacterium-specific imaging tracers are also being evaluated in clinical studies (71, 84, 85) and could help in establishing specific diagnosis of deep-seated bacterial infections that are not easily amenable to detection by traditional tools, monitor and prognosticate treatments, and optimize antimicrobial treatments. Early and specific detection of infections as well as dual-tracer imaging approaches that could provide accurate data on the class of bacteria causing the infections could help in streamlining empirical antimicrobial choices. Additionally, pathogen-specific tracers could also be used to specifically monitor the presence of viable bacteria and help in determining the treatment duration. Validated tools could also be utilized for precision medicine approaches for patients with complicated infections. Finally, since much of our understanding of infections is derived from animal studies or from biopsy specimen and tissue resection in humans, molecular imaging could enable basic research in humans. Multimodality imaging could simultaneously visualize many different processes (bacterial burden, antibiotic exposure, local microenvironment) and allow integration of cross-species data from animals to humans that is not feasible with current *ex vivo* tools.
